# Extracts

**Published:** 1883-02

**Authors:** 


### Extracts.

---

TUBERCULOSIS.—When the thermometer was first recommended as a diagnostic agent, it met with ridicule like every innovation. I remember the smile of derision with which the statement was made and received, that we might diagnosticate with it a case of typhoid fever. Contemptuous observations were not lacking of the credulity of a certain class of enthusiasts, who even maintained that a more accurate prognosis could be established by the thermometer than by the pulse, the abdominal symptoms, the facial expression, or that indefinable *tout ensemble* which was supposed to impress itself upon the older practitioner, whose knowledge was alone entitled to respect because it was based upon "experience."

The means of recognition of tuberculosis is now such a simple thing that there is no excuse for a mistake in diagnosis. I propose to speak of it now in full; and we will have the patient taken out, as his presence cannot aid us, and we have here in this sputum cup all we want for a diagnosis. But let me say something first of the steps which led up to this great discovery:

The gradual evolution of truth is rarely better exhibited in any field of our science than by a review of the principal points connected with the history of tuberculosis, the mystery of medicine, as it has been not inaptly called. The fact that one seventh of all mankind die of it, including one third of the productive population, and that most men are attacked by it at some period or other of their lives, as is evidenced by post-mortem examinations, has always surrounded the study of this disease with the very deepest interest. And the recent disclosures regarding the cause of it have made this interest in our day intense. Moreover, tuberculosis is a subject which concerns the surgeon, the obstetrician, the sanitarian, the specialist in whatever field, in almost equal degree with the physician. The pathologist has always regarded it as the foundation-stone in his science.

The new light in pathology, Cohnheim, illustrious through the comparatively trivial discovery of the emigration of the white blood corpuscles, objected to Villemin's conclusions, with the statement that he could produce the same results by the inoculation of other matter than caseous; in fact, by the inoculation of perfectly indifferent and inocuous matter, like elder pith, India rubber, etc. Thus the specificness of tuberculosis was lost again. But the doctrine would not stay down; on the contrary, it kept continually coming to the front. Next, 1878, came the simple experiments of Tappeiner, who found that dogs shut up in boxes and compelled to breathe atomized tuberculous sputa invariably contracted the disease, and with these simple experiments the doctrine of specificness was launched again, this time to remain upon the surface.

The observations of our own decade went with singular unanimity to confirm this view. Cohnheim was among the first to discover the sources of error in his previous experiments, and was the chief to reinstate the individuality of the disease. Not the giant cells, he put it; not the process of caseation; no other factor characterizes the disease except the capability of inoculation. The sole criterion of tuberculous matter is inoculability; and a whole host of ophthalmologists, headed by Harnsell, in in 1879, anticipated or confirmed this view.

Meanwhile, the pathologists in various fields had succeeded in establishing the identity of the various forms of tuberculosis. Thus, Koster showed that fungus joint affections were tubercular; Schuppel showed that scrofulous lymph glands were tubercular; Friedlander that caseous osteo-myelitis was tubercular, and Rindfleisch that all caseous pulmonary phthisis was tubercular. Pott's disease, certain affections of the skin, choroid, conjunctiva, cornea, iris, intima of the blood vessels, muscular coat of the uterus, were all quickly shown to be of the same nature, and thus the specificity of the disease was gradually put in an unassailable position.

It was the beginning of our century which witnessed the establishment of this fact. But such a doctrine could not sooner be established than search must have been instituted to discover its specific cause. Klebs, Schuller and Aufrecht claimed to have found a specific bacterium and micrococcus, but neither of them cultivated it or produced the disease. As we all know now, Koch did find it, describe it, cultivate it, and inoculate it in a series of experiments which will fix his name forever in the annals of medicine.

I shall not weary you by repeating again the details of the process by which the bacillus tuberculosis is made visible, nor the description of the bacillus itself. There is nothing new to add to either point. Nor has there been advanced a single objection as yet which is worthy of notice or will receive notice, I fancy, at the hands of the author of this discovery. For the statement of Moxon that the bacillus may be a product of the disease, like the spermatozoids of the testicle, is a fanciful conception; and the statement of Schmidt that the bacillus is only a fat crystal shows that he was dealing with pseudo-structures and not bacilli at all. Strange things turn out to be true sometimes, but the idea that we may be able to cultivate spermatozoids or like structures in a suitable soil does not accord with our knowledge of their development, and the fat crystal theory entirely ignores all culture and inoculation experiments in a way that is not creditable to the amenities of science, and does not hence merit notice. Moreover, fat crystals do not hold aniline dyes after immersion in nitric acid.

What is new about the bacillus tuberculosis is the fact that it can be found in all cases of pulmonary phthisis attended with expectoration. Blmumler and Fraentzel, who have just completed an examination of one hundred and twenty cases, declare that they never failed to find it in the sputum, and they never could find it in the non-tuberculous sputum; so that the bacillus has a higher diagnostic value than was at first believed. The bacilli are present in greater number in the sputum than in the lung structure (wall of cavities), proof that the sputum is a better nidus for it than living structure. Moreover, they are always present in greater numbers in the febrile cases than in those which run an afebrile course, or in the stage of pyrexia than in that of apyrexia in the same case. They accumulate by the myriad in cases near the end. It is in these bad cases that the bacilli have overflown the tissue of the lungs to inundate the whole body. It is the swift multiplication of the bacillus and irruption into veins that makes the case of "galloping consumption," that converts a "latent" into a "florid" case. Weigert, of Leipsic, has recently demonstrated all the steps of this process, the tuberculous nodules in the lining membrane of the veins, their erosion, and the "generalization" of tuberculosis by the blood.

Pretty much every case is difficult to diagnosticate at the very inception of the disease, because there is a stage of tuberculosis—I will not say phthisis, because the word, meaning only a symptom, ought to be abolished—which gives rise to no signs of any kind. Many a case so far advanced as to show hemoptysis has no other rational and no physical signs at all, and in all these cases we shall probably be able to make a diagnosis by looking for the cause of the disease.

Not only a diagnosis, but a prognosis too, can we make absolute by an examination of the sputum; for the number and development of the bacilli—that is, richness in spores—stand in direct relation to the progress of the disease. As the disease improves, the number of the bacilli diminishes, to disappear entirely when the patient recovers, or, what is more frequent, again becomes latent. In our case, as in every florid case, the sputum looks as if wholly compased of these structures, so myriad in numbers do they appear to be.

Of prophylaxis I will say nothing here, but it is plain to see that the day is not very far distant when we shall have the disease under control, not by attenuation of the vitality of the bacillus and its inoculation, but by preventing the dissemination of the disease. But that day is distant as yet, and it is especially far away because so many physicians are unwilling to accept the bacillus in its etiological relation to the disease until its acceptance is so universal that the newsboys and bootblacks on the street are familiar with the fact.

It was a long stride in advance when we got the thermometer to help us in a doubtful case, but it was a longer and a stronger stride when the higher powers of the microscope were invoked.—*James T. Whitaker, M.D., in Louisville Med. News.*

---

Bromide of Ethyl, the Most Perfect Anæsthetic for Short, Painful Surgical Operations.—Three years since, when the bromide of ethyl was brought prominently forward as a substitute for chloroform by Dr. R. J. Levis and Dr. Laurence Turnbull, both of Philadelphia, I, with other surgeons, experimented with the new anæsthetic, with the intent of comparing its reputed advantages with the well known agents sulphuric ether and chloroform. I discarded it after a very short trial on account of its apparent inefficiency, and because of the very evanescent nature of the sleep induced by it. I found great difficulty in putting my patients to sleep; and when at last narcotised, they would suddenly recover consciousness at most awkward periods in the midst of eye operations, to my serious annoyance. In two cases especially, in which I continued the inhalation from time to time as I would have done with chloroform, until upwards of an ounce of the bromide of ethyl was used, nausea and vomiting followed, which, in its severity and duration, I have rarely seen exceeded in the most sensitive of my chloroform patients. For twenty-four hours the sickness of stomach continued. The hospital ward in which the patients were lying had its atmosphere redolent with the garlicky odor of phosphorous, and the breath of these patients was offensive from the same smell on the day after the inhalation. For some months after this very unsatifactory, brief experience my bromide of ethyl bottle remained corked. About this time great publicity was given to a death in the practice of Dr. Marion Sims, from ethyl administration, in which several ounces had been used, and the narcosis kept up for a long period. This was the first time that ethyl had been inhaled for anæsthetic purposes in New York City. This first fatal case was followed soon afterwards by a death under the use of ethyl in the practice of Dr. Levis, of Philadelphia. These two fatal cases put a very sudden stop to the use of bromide of ethyl in the United States.

Confiding in the statements of Drs. Levis and Turnbull, both of them surgeons of large experience, that the bromide of ethyl had good properties, I was still disposed to believe that the new and comparatively unknown anæsthetic possessed attributes which we had not succeeded in developing. I therefore again commenced experimenting cautiously with this new remedy. By degrees, as I became better acquainted with it, it secured my confidence. For the past year I have used it on an average at least once every day, often administering it four, five, or six times, during the day's work in private practice and at the hospital. Familiarity with its peculiarities and effects, and the discovery of the proper method of administering it, has taught me to value its advantages more and more highly, till now I consider it *par excellence the anæsthetic* to be used for any painful surgical operation which can be quickly performed. Having found out how to use it, and what to expect from its administration, I can obtain the most brilliant results from it, and have become quite enthusiastic in its praises.

In every patient, using the needful precaution, I have produced complete narcosis in less than one minute, often, in from twenty to thirty seconds, a deep sleep which, however, will not last more than one or two minutes. From this speedily induced narcosis recovery is rapid and complete, with neither nausea nor heaviness, so that as a rule five minutes after the inhalation the patient is as much himself as if no anæsthetic had been used. Experience has taught me that these are the peculiarities of the bromide of ethyl when administered for anæesthetic purposes, and that as such they will prove of inestimable value to surgery.

Its wonderful action is obtained during the first minute of its inhalation and what I have called its primary anæsthesia.

In cases, in which from some interference with the rapidity of the manual of operative procedure this primary anæsthesia wears off, and a second, and even more numerous administrations have to be made to keep up the anæsthetic state until the operation can be completed, while the narcosis can at all times be reproduced, nausea is very apt to follow. By this frequent repetition of the inhalation, a mental depression is established, as from the continued use of chloroform or ether, which may last many hours.

Fortunately there are many surgical operations of a very painful nature which can be perfected within the short period of a primary ethyl narcosis. Abscesses can be lanced, cysts emptied, sinuses laid open, wounds probed, strictures incised, muscles divided, ingrowing nails removed, surfaces cauterized, examinations made necessitating painful manipulations, and even amputations may be performed. It must not be forgotten that prior to the discovery of anæsthetics, Mr. Lister urged the general adoption of flap amputations, because all painful cutting, including the sawing of the bones, could be completed in so many seconds and did not require minutes at the hands of dextrous surgeons.

In eye and ear surgery, in which I am now exclusively interested, the irritable eyes of children can be carefully and thoroughly examined, tumors can be removed from the lids, abscesses punctured, orbital sinuses explored, the lachrymal canals laid open, the nasal ducts probed, foreign bodies removed from the cornea, canthotomy practiced, crossed eyes straightened, the operation for artificial pupil perfected, ingrowing lashes destroyed by the cautery, needle operations for soft or capsular cataracts effected, and even optico-ciliary neurotomy completed. All such operations I perform now under a primary ethylization, if the patient exhibits any timidity or expresses a desire to be put to sleep. Cataract extractions, enucleations and many lid operations require more time for their safe performances than ethyl narcosis permits. If every preparation be made in advance, instruments arranged in the order in which they are to be used, and placed within easy reach, and if the surgeon is able to manipulate with dexterity, it can be readily seen that a very large part of the painful procedures of surgical practice might be made altogether painless by taking advantage of the wonderful nature of the ethyl narcosis.

In Eye Surgery I not only use ethyl daily, but if deprived of it would feel that I had lost one of my very best assistants.

What can be more satisfactory than the correction of that ugly deformity, squint, under the perfectly quieting influence of the bromide of ethyl, in less than one minute, to cover ethylization and the tenotomy? In fifty-two seconds, as measured by the stop watch, I have ethylized the patient and completed the division of the faulty muscle. The patient, quite himself in two minutes more, finds the ugly deformity gone, and without the slightest knowledge, on his part, of how the wonderful transformation has been brought about. This was my most expeditious operation. In the presence of the large medical class of the University of Maryland I have repeatedly completed the entire operation for the correction of squint, including the whole time necessary for the administration of the anææsthetic, in less than sixty seconds, as measured by the stop watch.

To use the bromide of ethyl efficiently, one must have confidence in himself and also in the safety of the agent which he is administering.

For long operations, or such as I desire to complete slowly, I prefer to administer chloroform, an anæsthetic with which I have had a long, extensive and uninteruptedly satisfactory experience. Of over 12,000 patients, upon whom I have operated under the narcotic effects of chloroform, I have not lost one. These patients cover organic disorders of heart, lungs, kidney or visceral disease, in persons of all ages, from the child only a few days old to my oldest chloroform administration, a very old man of ninety-six. Some were strong while others were very feeble. I never refuse the comforts of an anæsthetic to any person upon whom I have to operate.

Chloroform has always served me so faithfully that I have never had any good reason for transfering my allegiance to sulphuric ether. I now and then use ether but only at long intervals. Should a patient express any positive objection to chloroform and desire that ether be administered in his case, I always carry out his wishes. When the selection of the anæsthetic is left to me, and it usually is, my preference is decidedly for chloroform. I use chloroform so freely that I buy it literally by the gallon or in seventy-pound bottles, many of which I have emptied. Of sulphuric ether I still have a pound bottle which has been in my possession already five years, with contents not yet consumed. I believe that sulphuric ether is as safe as chloroform, but not more so. I know it to be more disagreeable in its odor and much more unpleasant in its inhalation. I believe that either chloroform or ether, when carefully given in accordance with well known laws, which should always be observed in the inhalation of anæsthetics, will with few very rare exceptions carry safety in its train. I also believe that if proper care be not taken, trouble may come to both patient and surgeon regardless of the agent selected. Some physicians have much more anxiety while using anæsthetics than others, not because they have a worse class of patients, but because they have never acquired the necessary confidence in the article they use, nor do they feel the necessity, under conviction, of always having and observing fixed rules for their guidance in the use of these powerful agents.

After an experience of thirty years of an active surgical practice, I still hold chloroform to be the best of anæsthetics for tedious operations, provided certain simple rules are adhered to in its administration. I can enumerate them in very few words:

1. I always, without a single exception, give a strong drink of whisky, from one to two ounces, to every adult to whom I intend to administer chloroform. This is done a few minutes before they get on the operating table. Because I never omit this fundamental law, and in advance sustain the heart against the depressing effect of the anæsthetic, in not one of my 12,000 cases have I had to use, in a single instance, a hypodermic of whisky. It is already in the stomach, should it be needed, and can do no harm if not required.

2. Always loose the neck and chest clothing so as to have no impediment to respiration.

3. Only administer chloroform in the recumbent posture with body perfectly horizontal and head on a low pillow, this pillow to be removed as the anæsthesia progresses.

4. Give chloroform on a thin towel folded in conical form with open apex so that the vapor, before inhalation, will be freely diluted with atmospheric air. In holding this cone over the face of the patient at some little distance from the nose, place the fingers under the borders of the cone for the double purpose of allowing air to enter freely, and also to prevent the chloroform liquid on the towel from coming in contact with the skin of the patient's face, and thereby avoid its blistering effects.

5. Should loud snoring occur, force up the chin. This manipulation, by straightening the air passages from the nose to the larynx, makes easy breathing. The forcible elevation of the chin is far better in every respect than pulling out the tongue. It is easier of application, more quickly done, requires no instruments, and is much more efficient in removing the impediment to respiration.

By always following these five simple rules I have had, so far, both safety and comfort in the administration of chloroform.

Possibly one very strong reason why I have been so successful in the administration of chloroform is, that as a specialist in eye surgery the inhaler must be removed from the nose before I commence the surgical manipulations. Besides, while operating, I have constantly in view both the color of the face and the respiration of the patient, which I consider even more important for the surgeon to observe than to feel the pulse. When surgeons are operating on distant parts of the body and cannot watch the administrator of chloroform, accidents are most apt to happen.

In the inhalation of the bromide of ethyl all of these rules laid down for the establishing of chloroform narcosis are not necessary, and some of them cannot be followed out.

The recumbent posture I consider essential for the safe administration of any anæsthetic, whether it be chloroform, ether or ethyl, hence these agents are not safe remedies in the hands of dentists, who place their patients in a sitting posture. Preparatory to the inhalation of the bromide of ethyl I have not found it necessary to give whisky. The only precaution I take is to loose the neck clothing and have the patient lie down with the head only slightly elevated.

My experience has taught me that the mode of administering the ethyl should differ totally from that used in giving chloroform.

Instead of a chloroform vapor freely diluted with atmospheric air, a saturated ethyl vapor must be inhaled, to the exclusion of atmospheric air, in order to obtain speedily and effectually narcosis.

In my early experiments with this new agent I had not yet discovered this fundamental principle, and hence did not obtain good results. I voted bromide of ethyl a failure, because in common with other experimenters, I was too timid, or rather I should say too ignorant of its peculiarities, to push the ethyl vapor in the concentrated form, which I have since found necessary to obtain good results. By my present method of administering it, I can obtain perfect ethylization in patients in from twenty to sixty seconds, and have no after consequences of nausea or dullness of feeling.

The best inhaler for the giving of the bromide of ethyl is a thick towel folded into the form of a small cone with closed apex. Between one of the folds of the towel I place a sheet of paper, which makes the cone nearly air tight. The base of the cone must be wide enough to enclose both mouth and nose. The soft material of which the inhaler is made enables the rim to be kept firmly in contact with the face, so as to exclude air from entering. I always instruct the patient how to make long inspirations, and inform him that he must do this, notwithstanding the fact that he will feel somewhat stifled. I also try to give him confidence by assuring him that a very few inspirations will put him to sleep. Usually I make him go through the process of strong respiratory movements in advance, so that he will know exactly how to proceed. Into this towel cone I pour about one drachm of the bromide of ethyl and immediately invert the inhaler over the nose and mouth of the patient, holding its edge down firmly over the face. There is no fear of creating asphyxia, as all air cannot be excluded, and the height of the cone makes a considerable air chamber into which the patient breathes.

Children usually struggle to escape from the apparatus.

The cone, however, must not be removed from the face for an instant until anæsthesia is produced. At first some patients will resist the breathing of the vapor, but there is no fear that they will not catch their breath in time. Should children cry, it only insures inspiratory efforts, which the more surely and quickly will bring about the introduction of the vapor into the lungs. As a rule, a dozen full inspirations are all that are needed to produce deep narcosis. I recognize this desirable condition by a stoppage of all struggling. I have had deep sleep brought on by the sixth inspiration, when complete relaxation ensues, with quiet breathing, and an absence of reflex irritation should the conjunctiva be touched. The patient retains the usual healthy color of lips and cheeks as if in ordinary sleep, and the pulse becomes slower and stronger as the narcosis becomes profound. Thirty seconds, as a rule, is sufficient to bring about this desirable condition, and have the patient ready for operation.

I have not found this anæsthetic sleep last more than two or three minutes, often not so long.

Usually the patients awake suddenly and as completely as they would do from ordinary sleep. They are able to get down from the operating table without assistance and walk off without staggering, and with brain clear to answer correctly any question: in fact, quite themselves.

It took me some time to acquire such confidence in the safety of the remedy as to apply it in the concentrated form needful to obtain its fullest benefits. To the uninitiated it looks like cruel work to keep the cone of a saturated ethylized vapor over the face of a struggling patient. I am convinced, however, that in no other way can quick, complete and safe anæsthesia be obtained by it. Fortunately the struggling is very soon over, and quiet sleep speedily ensues.

My experience with the bromide of ethyl will now exceed 400 cases, of which upwards of 300 are within the past year. I am beginning to be familiar with its administration and its effects. I now know what is to be obtained by it, and what not to expect from it. I give it without hesitation, in any case, to avoid painful manipulation. I have used it as often as six times a day, and I administer it, on an average, certainly once every day. In the last week I have given it fifteen times. For office use I find it invaluable, on account of its promptness, efficiency, evanescent nature of the anæsthesia induced, the absence of nausea, and the perfect comfort with which patients operated upon can leave my office within a few minutes after the ethylization. Its use in my every-day experience does not interfere with the routine of office practice, nor occupy more time than I give to an ordinary office consultation, a very important desideratum to those who have restless patients awaiting their turn in the reception room.—*Julian J. Chisolm, M.D., in Maryland Medical Journal.*

---

Ladies' Doctors.—The decay of the curate and his supplantation by the ladies' doctor, is the subject of a very clever and witty sketch which appeared recently in the London *World.* The writer's portrayal of the modern ladies' doctor is not in every point flattering, but there is much truth in it, and it is well to have a mirror held up before us occasionally. The day in which the curate ruled the drawing-room in its less intellectual corners is, says the *World*, rapidly passing away. Coincident with his decay is the rise in popularity of the ladies' doctor. This new luminary is a modern institution. A century ago, to be sure, says the writer, incomes as large as any of to-day were made by fashionable physicians, but these physicians were only "medical men," and held no social sway. "Astley Cooper and Abernethy were surgeons of weight and even wit, but their *brusquerie* made them more the terror than the darlings of a drawing-room, and men of their type were no more welcome in their non-professional capacity than are the plumbers after they have soldered a leaky pipe."

The writer follows with a sketch of what constitutes, or should constitute, the successful ladies' doctor of to-day —a person who is called upon to minister to weak nerves, feeble livers and the other incidentals to late hours and much caring for many things.

He must, we are told, be a skilful physician, perhaps, but certainly, and above all things, he must be a man of the world. "He must know the ways and weaknesses of his clients, and not dream of playing the part of their moral instead of their medical mentor. Hence he is familiar with all the topics of the day, and yet is not pronounced on any one of them, for he is too wise to offend the patients by whom he thrives. He may know the winner of the Derby, but he must not be seen at Ascot and Epsom, and, though his coachman may drive the best of blood, it would ruin him were it reported that he had been seen behind a four-in-hand. He may write medical works—the more the better—but society is skeptical regarding omniscience. Sir Charles Bell's popularity fell off after he published his book on the hand. Yet art, being only an elegant amusement, may be pursued. Sir Henry Thompson collects china and pictures, and Mr. Seymour Hayden etches, while a good many doctors garden, as did Sir William Lawrence, without finding their practice suffer. The ladies' doctor must not be an atheist —society has not come to that yet, and women who are nervous are always pious—but unless he cultivates a Dissenting connection, experience proves that it is safest for him to keep to a strictly orthodox course, neither too broad church, nor yet with a suspicion of incense and the eastward position. For ladies with a taste for ritualism instinctively lean on the curate, and between him and his supplanter there can never be anything in common."

The above sketch shows only what every one learns very soon—that in order to be a successful and especially a fashionable physician, tact and wordly knowledge are the essentials; knowledge and skill are only useful supplements. Too much knowledge, especially if it be of a more purely scientific character, is likely, in this country, to call down the reproach that its possessor is not "practical." To offset the ladies' doctor of to day, however, we have the specialist, who can generally be as scientific and unwordly as he pleases.—*N. Y. Med. Record.*

---

Carbolic Acid in the Treatment of Wounds.—I will state, at the outset, my decided conviction that the use of carbolic acid is a valuable adjuvant in surgical practice. As a mere disinfectant, carbolic acid seems to me to be in no way superior to a number of other agents, but probably inferior to some of them; as an antiseptic and detergent, however, I am inclined to believe that it is not surpassed by any other substance. As to its supposed specific property of allaying or antagonizing the inflammatory process, apart from its antiseptic action, my impression is that the stock of well ascertained facts at our command is too small to warrant our drawing any decided conclusion. It is to its germicidal properties, in my opinion, that its efficiency in averting inflammatory complications is mainly due. It is valuable also as a topical stimulant to ill-conditioned suppurating surfaces, such as the walls of cold abscesses and the like, in patients broken down in health.

As a matter of course, a germicide is needed only under circumstances that make it a reasonable inference that morbific germs are actually present in the air about the patient, or in the implements and appliances that are used in dressing the wound; carbolic acid, therefore, is of the most signal advantage in the case of patients who have to be treated in close, ill-ventilated rooms, or within the range of exhalations from diseased surfaces in other patients—or, in general terms, under any circumstances in which the sufferer is compelled to breathe a confined atmosphere, perhaps contaminated with morbid animal effluvia.

On the whole, it does not yet appear to me that operative surgery, leaving out of account those operations in which the abdominal cavity is opened, is more successful under Lister's method of practice than it used to be before that system came into use; certainly, in the case of clean wounds, well cared for and treated in the country, it is my decided impression that no better results can be got with Listerism than without it. In saying this, I do not lose sight of the fact that, no matter how favorable the surroundings may be, pent-up and putrescent material involves terrible dangers. Under such circumstances carbolized injections are unquestionably of the greatest value, but from this point of view there is nothing new about antiseptic surgery; in former times the chlorine preparations and other detergents and antiseptics doubtless proved equally efficient preventives of septicæmia.

Like everything else that has the charm of novelty, the use of carbolic acid has been pushed, I believe, beyond the limits that will in the future be set upon it; at present the strict measure of its value does not seem to me to be fully settled, nor has it been quite determined under what circumstances it should or should not be resorted to. I am not yet convinced that its employment is imperative as a routine measure, but am quite willing to concede to it the character of a most valuable resource in cases that plainly call for it.—*Willard Parker, M.D., in New York Med. Journal.*

---

Treatment of Neuralgia.—With regard to the various anti-neuralgic remedies in general use, their efficacy consists for the most part in diminishing, or altogether removing, the pain for the time. They are essentially pain-killers. Many of them are simply local applications—as ointments of atropia, aconite, or veratria; oil of peppermint; a combination of iodoform and chloroform; chloroform alone; chloral and camphor; liniment of belladonna; a strong infusion of capsicum on lint, covered over with gutta-percha; ether spray; hydrocyanic acid and cyanide of potassium; oleate of morphia, etc. Sometimes the benefit of the remedy—taken internally—is limited to certain nerves—as chloride of ammonium, valerianate of zinc, chlorate of potash, croton chloral, etc., in facial neuralgia; gelseminum, chamomile, nitrite of amyl (inhalation), for neuralgia of the fifth pair; carbonic acid injection for neuralgia of the uterus. Then, various remedies are employed to diminish the sensibility of the nervous system generally—as bromide of potassium, cannabis indica, conium, stramonium inhalation, the spinal ice-bag, etc., etc.; or to give that to the nervous system which is supposed to be deficient—viz.: phosphorus; or to stimulate it—as tea, coffee, guarana; or to supply some deficiency in the blood—as iron in anæmia. Other remedies are prescribed to give tone to the nervous system and improve the general health—as electricity and galvanism, quinine, and arsenic; the two last, in cases of well-marked periodicity, being given in larger doses to anticipate an attack. Counter-irritation, in the form of flying blisters, has been recommended for the shifting neuralgic pains common in nervous sensitive women; but, as the pain is pretty sure to go elsewhere, and much cutaneous tenderness is often left, their value is doubtful. Flying blisters, in my own experience, are most beneficial where there is effusion between a nerve and its sheath, or thickening of the latter. I have found them of excellent service in the latter condition in sciatica, applied above, and in the course of, the seat of pain. They are also decidedly useful in intercostal neuralgia after shingles.

All these various remedies (and indeed in some instances nothing more is required—I refer particularly to those that are merely pain-killers) are of more or less value so far as they go; but, for the permanent cure of neuralgia, something more, generally speaking, *is* required. As before observed, all cases cannot, as a rule, be treated exactly alike. To begin with (this *is* a rule of almost universal application), I recommend total abstinence from alcoholic drinks, whether as beverages or medicine—as medicine, I mean, for the neuralgia. Let the neuralgic sufferer substitute Kepler's malt extract, or maltine, for his beer or stout, or even take no substitute, and he will be surprised at the result. It may be that, without anything further, the attacks of pain will altogether cease. If medicine be required, I strongly recommend the union of a nervine tonic and an anodyne, administered on a systematic principle, and to serve as a fortifier and tranquillizer of the weakened and irritable nervous system; and, when necessary, in still larger doses as an anti-periodic. In malarious fevers, by adding an opiate to the tonic (quinine), an earlier and more complete cure is insured. Less quinine, too, when that is given, is needed; and there is, consequently, less probability of tinnitus.

It is by no means necessary in all cases to give heroic doses of the anti-periodic; though, in those of a chronic nature, it will usually be necessary. Quinine, where admissible, is undoubtedly the best; but, when it cannot be borne, sulphate of cinchonine in similar doses answers almost as well. Salicine may be given in gr. x. doses, repeated at intervals of half an hour, up to gr. xxx. Neither of these drugs cause tinnitus when given, even in anti-periodic doses, unless, indeed, these be very large or frequently repeated. Where quinine can only be borne in moderate doses, it and arsenic go very well together; gr. v. of the former, and *m*x. of Fowler's solution; or, *m*xx. of this solution alone will suffice. Though, from ample experience with this drug, I should not hesitate to prescribe even so much as *m*xxx., and repeat it in a short time—say an hour—if absolutely necessary, I think it is preferable to use other anti-periodics in the first instance. Whenever arsenic *is* used, an anodyne should, of course, be combined with it, to counteract its irritating action on the stomach and intestines. It is well to bear in mind that quinine may often be tolerated hypodermically, when the stomach will not bear it, and that gr. v., thus introduced, is equal in efficacy to gr. xxx. swallowed. The neutral sulphate should be used. It is readily soluble (gr. j. to *m*xij.) in water without the aid of any acid—a great advantage, as there is then less fear of irritation of the skin, and boils. It is not uncommon for a patient to say, on hearing that quinine is to be given: "Oh! I've taken lots of quinine." Yes, but how? One frequently finds it prescribed in small doses as a general tonic, but rarely, except, of course, in well defined malarious cases with periodicity, as an anti-periodic. Neuralgic patients often require a deal of looking after. And herein lies the advantage which the practitioner, who sees his patient almost daily, enjoys over the physician who perhaps has only seen him once.

The *nature* of the tonics is a point for consideration. Easton's syrup, as containing quinine, iron, strychnine and phosphorus, is the best where the several ingredients can be borne. But, whatever be selected, I must repeat my recommendation that an anodyne be combined with it, care being taken to counteract any functional irregularities that may be caused by its exhibition. And of anodynes I would advocate nepenthe, a preparation of opium manufactured by Messrs. Ferris & Co, of Bristol, which deranges the functions, if at all, less than any other—resembling in this respect the bi-meconate of morphia. Hyoscyamus may be substituted in the case of those who cannot bear opium in any shape; or conium, or belladonna, or croton chloral. This last is very promising. Belladonna, which we find in India to be so serviceable in alleviating the neuralgic pains of dengue, might be more extensively used.

Of surgical remedies, as nerve tapping, stretching, and the like, I have no personal experience. One can readily understand that the victim of neuralgia in its most agonising forms—*e.g.*, tic douloureux—would readily hail any measure that gave promise of relief, even excision of a ganglion. There are not cases enough of this operation, or of nerve stretching, on record from which to form an opinion as to the efficacy of either; but, inasmuch as sensations—pain, even—have been felt in the distal ends of an extremity after removal of the limb, I should question the permenent value of the former; and, notwithstanding the recorded efficacy of the latter, I should be too afraid of tetanus (a result which *has* indeed occurred) to submit to it myself. It seems, moreover, so directly opposed to that most valuable of all remedies, and from which the nervous system would specially derive advantage—viz, *rest.* I once had a patient who declared that he constantly had neuralgia—and very severely, too—in his *hair!* Such a case would surely be beyond the reach of surgical interference!

Amongst the most troublesome of the so-called neuralgic cases that come before us are those where neuralgia seems to be blended with myalgia. I am acquainted with a lady who declares that she has a cancer growing in her right side—the exact spot being about the centre of the infra spinatus muscle. The pain, which is always referred to the same spot, is not constant; indeed, she is sometimes without it for several days. It is unaffected by pressure, and unaccompanied by swelling; nor is there any sign whatever of malignant disease. Playing the piano brings on the pain after a time.

In some of these cases there is a rheumatic basis, as in the omodynia, scapulodynia, and dorsodynia, observed amongst laboring men; in others there may, at some previous period, have been slight rupture of muscular fibres; in others there may be a neuralgic origin; in all, more or less defective innervation, so that the particular muscle affected is easily fatigued—pain even being sometimes induced—on exertion. Each class of cases must, of course, be treated in part according to its original nature. A mixed soothing and stimulating plan will usually be attended with most success; but, speaking generally, many cases of this description are, I venture to think, well suited for treatment by massage. To the limited extent of shampooning, or muscular kneading, practitioners in India are familiar enough with massage. No one who has not experienced it can appreciate the effect induced, where the tired muscles of the legs are kneaded by the hands of an expert after a day's shooting, or other hard work. Fatigue is no longer felt to the extent of preventing sleep, but, in its place, a sensation of pleasuable weariness that cannot be described; nay, sometimes, the weariness disappears altogether as if by magic.

It may be sound and very common sense advice to give to a lady patient, who complains that when she crochets she has pain in one particular spot in her side—"Don't crochet." But something more would be expected from a physician; and I believe that in massage we possess an excellent remedy that is well suited to these cases, where the uneasiness—it often hardly amounts to pain—constantly returns in the same spot, until at last the mind is not unnaturally led to imagine the worst. In addition to the general treatment suited to the particular case, whatever amuses the mind and, lifting it as it were out of itself, diverts it into other channels, must have a beneficial effect.

Neuralgia is often regarded as hopelessly incurable. And so, at one time, was the "collapse" condition in cholera; whereas, now, fifty per cent., under appropriate treatment and if the patient have a good constitution, recover. I would, therefore, urge upon my professional brethren, when treating the most unpromising case of neuralgia as when endeavoring to maintain life in the most advanced stage of that fell disorder, the adoption of the motto "*Perseverando.*"—*Charles R. Francis, M.B., Lond., in Med. Press and Circular.*

---

The Treatment of Rheumatism by Blisters.—About twenty-five years ago I had the opportunity of studying the treatment of rheumatic fever by two physicians in the same wards. One of them prescribed opium freely, and the other gave no internal remedies; both had the affected joints carefully enveloped in cotton-wool, as they became attacked, and then firmly bound with flannel bandages, free perspiration being encouraged also by blankets, etc. The result seemed to me to be much the same: that the affected joints rapidly ceased to be painful, and that the patients made, as a rule, a good recovery.

Since that time my treatment of rheumatic fever has kept very much in those lines (with, in some cases, the addition of salicin); and though I have seen many methods of treatment tried, and much extolled, I cannot say that they have appeared to me to have any special recommendations. But against one treatment I cannot resist, at the present moment, raising my humble protest, viz., the "garter blister," as again advocated by Dr. Davies. When so eminent a physician, with all the authority of long experience of a treatment originated by himself, can still advocate it, he must be very confident of its specially beneficial action, and no doubt must have many followers; but of all treatments (except packing) it is the one which I would not have tried upon myself. I had quite hoped that, long ere this, it had died a natural death. I have seen patients suffer such great discomfort from the raw surfaces of these circular blisters above the knee-joints when other joints have become affected, as to make them beg not to have any more applied. I ask, therefore, why should patients be given this additional suffering as a local treatment, when rapid freedom from pain can almost certainly be obtained by careful bandaging, as above stated; and if it be intended as a constitutional treatment, why choose such a specially painful form of blister as the "garter"?

It is some time since I have been able to watch closely this circular blister treatment; but it made a very decided impression upon me that it did not cut short the attack, and that it was very objectionable from the patient's point of view.—*A. B. R. Myers, M.R.C.S., Eng., in British Med. Journal.*

---

TRACHEOTOMY.—From the experience of others and from my own, I have arrived at the following conclusions:

1. The point of election is just below the cricoid isthmus.

2. The isthmus of the thyroid, if recognized, should be pushed down, the cervical fascia of the median line having first been incised, and the trachea exposed by carefully separating the parts with a director; or,

3. The thyroid isthmus may be entirely disregarded, and the parts freely incised, in which case all hemorrhage should be checked before opening the trachea.

4. Deliberation, careful dissection and a bloodless operation is better than the gain of a few seconds at the expense of hemorrhage into the trachea.

5 Ether should be used except in extreme asphyxia.—*M. H. Richardson, M.D., in Boston Med. and Surg. Jour.*

---

NYMPHOMANIA AND HEMORRHOIDS.—At the last meeting of the Medical Society of the State of Arkansas, Dr. William H. Hardison related a case of nymphomania, which caused a respectable young married woman to masturbate and to so deport herself as to cause the greatest family unhappiness. All drugs were powerless so relieve her. It was finally discovered that she was afflicted with very sensitive hemorrhoids, fissure of the anus and constipation. Operation and attention to the bowels so thoroughly relieved her that she almost went to the other extreme.—*Med. and Surg. Reporter.*

